# Editorial: Molecular interactions between crops and phytopathogens volume III: Vegetables and other crops

**DOI:** 10.3389/fpls.2022.979342

**Published:** 2022-07-27

**Authors:** Meixiang Zhang, Xiao-Ren Chen, Xiaojie Wang, Guotian Li, Lisong Ma, Jin-Ying Gou, Jianhui Wu, Xiaodong Wang

**Affiliations:** ^1^National Engineering Laboratory for Endangered Medicinal Resource Development in Northwest China, Key Laboratory of Medicinal Resources and Natural Pharmaceutical Chemistry of Ministry of Education, College of Life Sciences, Shaanxi Normal University, Xi'an, China; ^2^College of Horticulture and Plant Protection, Yangzhou University, Yangzhou, China; ^3^State Key Laboratory of Crop Stress Biology for Arid Areas, College of Plant Protection, Northwest Agriculture and Forestry University, Xianyang, China; ^4^State Key Laboratory of Agricultural Microbiology and Provincial Key Laboratory of Plant Pathology of Hubei Province, College of Plant Science and Technology, Huazhong Agricultural University, Wuhan, China; ^5^State Key Laboratory of North China Crop Improvement and Regulation, College of Horticulture, Hebei Agricultural University, Baoding, China; ^6^State Key Laboratory of Genetic Engineering, MOE Key Laboratory for Biodiversity Science and Ecological Engineering, MOE Engineering Research Center of Gene Technology, Institute of Plant Biology, School of Life Sciences, Fudan University, Shanghai, China; ^7^College of Agronomy, Northwest Agriculture and Forestry University, Xianyang, China; ^8^State Key Laboratory of North China Crop Improvement and Regulation, College of Plant Protection, Hebei Agricultural University, Baoding, China

**Keywords:** crop-pathogen interactions, genome-wide identification, jasmonic acid signaling, pathogenicity-related genes, biocontrol

Plant diseases cause substantial annual yield losses of crops, and pose a major threat to global food security and agricultural sustainability. Improving crop resistance against diverse diseases plays a crucial role in safeguarding sustainable crop production to nourish the increasing world population. Dichperhing the molecular mechanisms underlying the interactions between crops and phytopathogens will provide a valuable basis for the improvement of crop resistance and disease management.

In the context of long-term coevolution, crops have developed sophisticated strategies to cope with phytopathogens. Invading phytopathogens, on the other hand, have evolved various defensive mechanisms to facilitate their invasions and infection. Understanding different crop-pathogen interactions will broaden our knowledge toward the dynamic coevolutionary arm races engaged by plants and pathogens. In this context, we organized this Research Topic on “Molecular Interactions between Crops and Phytopathogens Volume III: Vegetables and other crops.” Besides the three major food crops in the world, this Research Topic mainly focused on the valuable vegetables and other crops. This topic was presented by ten outstanding articles, which cover cabbage (Liu et al.), bell peppers (Yang et al.), litchi (Li et al.), tobacco (Yang et al.), *Brassica napus* Canola Adhikary et al.), barley (Yuan et al.; Xu et al.), rose (Tian et al.), soybean (Feng et al.), and a new model plant within the family Gramineae, *Brachypodium distachyon* (Peng et al.). According to the research content and research perspective, we broadly divided these ten articles into the following four themes:

## Identification of key genes involved in crop-pathogen interactions by omics approach

Technical breakthroughs in sequencing technology, and the rapid development of bioinformatics, including multi-omics tools, offer many new clues for understanding the molecular mechanisms of crop-pathogen interactions. The TRICHOME BIREFRINGENCE-LIKE (TBL) family participates in the O-acetylation of cell wall polysaccharides. Tian et al. profiled *TBL* gene family in rose genome and explored their functions during plant resistance to gray mold. Twelve of 50 *RcTBL* genes were down-regulated upon *Botrytis cinerea* infection, and knocking down of *RcTBL16* significantly enhanced plant resistance to *B. cinerea*, highlighting the importanceof the function of TBL proteins for future studies. Plant-specific lateral organ boundaries domain (LBD)-containing transcription factors are involved in plant responses to various stresses. Feng et al. investigated the *LBD* gene family in soybean on a genome-wide scale, and identified differentially expressed *LBD* genes upon *Phytophthora sojae* infection. They further showed that *GmLBD9* and *GmLBD23* negatively regulate plant defense against *P. sojae*, whereas *GmLBD16* and *GmLBD88* contribute to soybean defense against *P. sojae*. This study expands our knowledge about the origin and evolution of *GmLBD* gene family in soybean and promotes the potential application of these genes in disease resistance improvement. MicroRNAs (miRNAs) are one of the key components that control the transcriptional responses of plant to pathogen infection. Peng et al. identified the differentially expressed miRNA and their target genes in *Brachypodium distachyon* upon infection by *Magnaporthe oryzae*, providing an unraveling complex miRNA-mediated regulatory networks during *B. distachyon*-*M. oryzae* interactions. A NAC transcription factor *BdNAC21* gene was validated as a target of the differentially expressed *miR164c*. Identification of differentially abundant proteins (DAP) using proteomics reveals their direct functional role in plant-pathogen interactions. Adhikary et al. explored the DAPs associated with *Plasmodiophora brassicae* resistance in *Brassica napus* canola at different infection stages. Seventy-three DAPs annotated as orthologs to clubroot-resistant proteins and eight quantitative trait loci (QTLs) were identified, providing potential candidates conferring immune response to *P. brassicae* in canola.

## Characterization of pathogenicity-related genes in crop pathogens

Litchi downy blight caused by *Peronophythora litchii*, an oomycete pathogen, is a major disease in litchi. Cytochrome b_5_, an electron transport component, is essential in the Class II cytochrome P450 monooxygenation system. Li et al. identified a Cyt-b_5_ domain protein, PlCB5L1, in *P. litchi* and, for the first time, reported that this cytochrome b_5_ superfamily member contributed to the mycelial growth, stress response, and pathogenicity in *P. litchi*. The P4-ATPases, aminophospholipid translocases (APTs), play essential roles in the growth and pathogenesis of fungal pathogens. Yang et al. identified a P4-ATPase Drs 2 homolog PcApt1 in *Phytophthora capsici*, and demonstrated that PcApt1 participated in phosphatidylserine (PS) transport across the plasma membrane, the hyphal growth, extracellular laccase activity, and *P. capsici* virulence. Since both PlCB5L1 and PcApt1 are well conserved in oomycetes, these two studies provide new insights into the development of plant pathogenic oomycetes and hence are helpful for the control of related diseases. Powdery mildew, a biotrophic pathogen, secretes various effectors to manipulate plant cell death. Yuan et al. discovered a secreted effector protein CSEP0027 from the barley powdery mildew pathogen *Blumeria graminis* f. sp. *hordei* (*Bgh*) as a cell death inducer. CSEP0027 contributed to *Bgh* virulence in barley by directly interacting with barley catalase HvCAT1 and altering its subcellular localization, indicating that powdery mildew pathogen promotes its virulence by manipulating reactive oxygen species homeostasis during infection.

## Jasmonic acid signaling in crop-pathogen interactions

The plant hormone jasmonic acid (JA) plays essential roles in many biological processes, including plant defense against pathogens. Xu et al. explored transcriptomic, proteomic and metabolomic data of powdery mildew (PM)-susceptible and PM-resistant accessions to systemically examine the mechanisms of PM resistance. They found that the resistance to PM in qingke, also called “naked barley,” involves the accumulation of aromatic phenolamides through jasmonate-mediated activation of defense-related genes. N-acyl-homoserine lactones (AHLs) are the most common signal molecules in Gram-negative bacteria. Liu et al. found that AHL improved the resistance of Chinese cabbage and *Arabidopsis* to the hemibiotrophic bacteria *Pectobacterium carotovorum* ssp. *carotovorum*. In this process, the JA signaling pathway participates in AHL priming by coordinating with the auxin signaling pathway.

## Characterization of the Nep1-like proteins from biocontrol agent *Pythium oligandrum*

Necrosis and ethylene-inducing peptide 1-like proteins (NLPs) exhibit dual functions in plant-pathogen interactions, acting as both toxin-like virulence factors and triggers of plant immune responses. Yang et al. showed that the cytotoxin and immunity induction activity of NLPs are largely divergent. Both non-cytotoxic and cytotoxic PyolNLPs from the biocontrol agent *Pythium oligandrum* enhanced plant resistance to a wide range of pathogens. In addition, they found that the conserved nlp24-like peptide pattern is required for this process uncoupled with reactive oxygen species and cell death.

In summary, the collected articles in this Research Topic identified some key genes or pathways involved in crop-pathogen interactions, characterized their functions, and preliminarily explored their functional mechanisms. Data in these articles provide valuable gene resources for controlling crop diseases and further dissecting the molecular mechanisms underlying crop-phytopathogeninteractions.

## Author contributions

All authors have made a substantial and intellectural contribution to the work and have acted as co-editors of this special issue.

## Funding

MZ was supported by the National Natural Science Foundation of China (32072399, 31672008) and the Fundamental Research Funds for the Central Universities (GK202201017). X-RC was supported by the National Natural Science Foundation of China (31871907, 31671971) and Jiangsu Agriculture Science and Technology Innovation Fund (JASTIF) [CX(20)3125]. XW was supported by the Shaanxi Innovation Team Project (2018TD-004XW). GL was supported by the National Natural Science Foundation of China (32172373). LM was supported by the Hundred Talents Program for the introduction of high-level overseas talents in Hebei Province (E2020100004). JY-G was supported by the National Natural Science Foundation of China (31972350). JW was supported by the Key R&D Program of Shaanxi Province in China (2021ZDLNY0-01). XW was supported by the Provincial Natural Science Foundation of Hebei (C2022204010 and C2021204008) and Independent Project of State Key Laboratory of North China Crop Improvement and Regulation (NCCIR2021ZZ-4).

## Conflict of interest

The authors declare that the research was conducted in the absence of any commercial or financial relationships that could be construed as a potential conflict of interest.

## Publisher's note

All claims expressed in this article are solely those of the authors and do not necessarily represent those of their affiliated organizations, or those of the publisher, the editors and the reviewers. Any product that may be evaluated in this article, or claim that may be made by its manufacturer, is not guaranteed or endorsed by the publisher.

